# Enhanced Pro-Osteogenic Regulatory Modulation in Mesenchymal Stem Cells Derived from the Periosteum Under Simulated Microgravity

**DOI:** 10.3390/cells15110989

**Published:** 2026-05-28

**Authors:** Raul Canal, Elizabeth F. Martinez, Jamie S. Foster, Fernanda Carla Bombaldi de Souza, Renata Francielle Bombaldi de Souza, Marcelo Marcos Morales, Marcos Cesar Pontes, Daniel N. da Rocha, Lexie S. Holliday, Fahong Yu, Anderson Tadeu Silva, Roberto D. Fanganiello, José Ricardo M. Ferreira, André A. Pelegrine

**Affiliations:** 1 ANADEM (Sociedade Brasileira de Direito Médico e Bioética), Setor Shs, Quadra 2, Bloco J, Mezanino-Asa Sul, Brasilia 70322-901, Brazil; presidencia@anadem.org.br; 2 Division of Oral Pathology and Cell Biology, Faculdade São Leopoldo Mandic, Rua Dr. José Rocha Junqueira 13, Campinas 13045-755, Brazil; elizabeth.martinez@slmandic.edu.br; 3 Space Life Science Lab, Kennedy Space Center, Department of Microbiology and Cell Science, University of Florida, 505 Odyssey Way, Merritt Island, FL 32953, USA; jfoster@ufl.edu; 4 R-Crio Criogenia S.A., Rua Cumarú 204, Campinas 13098-324, Brazil; fernanda.bombaldi@r-crio.com (F.C.B.d.S.); renata.bombaldi@r-crio.com (R.F.B.d.S.); navarro@r-crio.com (D.N.d.R.); josericardo@r-crio.com (J.R.M.F.); 5 Institute of Biophysics, Federal University of Rio de Janeiro, Avenida Carlos Chagas Filho, 373-Bloco G, Rio de Janeiro 21941-902, Brazil; mmorales@biof.ufrj.br; 6 Technological Institute of Aviation, Praça Marechal Eduardo Gomes 50, São José dos Campos 12228-900, Brazil; 7 Department of Orthodontics, University of Florida, 1600 SW Archer Rd, Gainesville, FL 32611, USA; sholliday@dental.ufl.edu; 8 Interdisciplinary Center for Biotechnology Research, University of Florida, 2033 Mowry Road, Gainesville, FL 32610, USA; fyu@ufl.edu (F.Y.); anderson.silva@ufl.edu (A.T.S.); 9 Faculty of Science and Engineering, Université Laval, Pavillon Alexandre-Vachon1045, Avenue de la Médecine, Room 1033, Quebec City, QC G1V 0A6, Canada; robertofanganiello@gmail.com; 10 Division of Implant Dentistry, Faculdade São Leopoldo Mandic, Rua Dr. José Rocha Junqueira 13, Campinas 13045-755, Brazil

**Keywords:** osteogenesis, periosteum, osteogenic cells, simulated microgravity, gene expression, secretome

## Abstract

**Highlights:**

**What are the main findings?**

- Periosteum cells increased the production of important factors for bone formation in simulated microgravity
- Genes upregulated under simulated microgravity conditions in periosteum cells are related to osteogenesis

**What are the implications of the main findings?**

- The secretome of periosteum-derived cells cultured under simulated microgravity might be used to treat low bone density conditions
- The secretome of periosteum-derived cells cultured under simulated microgravity might be used to treat critical bone defects

**Abstract:**

This study aimed to evaluate periosteum-derived mesenchymal stem cells (P-MSCs) cultured under simulated microgravity (SMG) conditions. P-MSCs were induced toward osteogenic differentiation and then exposed to SMG for up to 48 h. As a control, P-MSCs were maintained under identical conditions but without SMG exposure. Cell viability, osteogenesis-related analytes, and gene expression were analyzed at 3, 24 and 48 h. Cell viability under SMG was lower after 3 h but was significantly higher after 24 h, with no difference at 48 h. There was a higher expression of pathways associated with inflammation at 3 h, which was attenuated by 24 h and neutralized at 48 h. P-MSCs under SMG demonstrated three characteristics in at least one timepoint, which supports a pro-osteogenic signaling response: (1) higher osteoprotegerin levels; (2) lower DKK1 and TNF levels; (3) upregulation of genes related to osteogenesis. Our data suggest that P-MSCs exhibit enhanced pro-osteogenic regulatory modulation in SMG.

## 1. Introduction

Periosteum is a tissue that covers the outer surface of bones and serves as a transition zone between cortical bone and the surrounding soft tissues and muscles. It contains osteogenic cells that regulate the external shape of the bone and coordinate with the inner cortical endosteum to control cortical thickness, as well as bone size and spatial positioning [1]. Periosteum-derived mesenchymal stem cells contribute to bone regeneration and have been suggested to be superior to bone marrow-derived cells in this regard [2]. Recently, Sanchez et al. [3] showed that human periosteum-derived cells demonstrated higher mineralization capabilities than human bone marrow-derived mesenchymal stromal cells. Moreover, periosteum is the primary source of callus formation during bone healing [4] and, due to its high vascularization, harbors many endothelial pericytes [5].

According to Pranskunas et al. [6], proteins secreted by periosteum-derived mesenchymal stem cells can stimulate osteogenesis, neurogenesis, and angiogenesis as well as have immunological properties. Bioceramic xenografts implanted with proteins secreted by periosteum-derived mesenchymal stem cells were found to enhance the formation of new bone. These cells have been isolated from several anatomical locations, including the knee [7], mastoid bone [8], tibia [9], femur [10], and various oral sites [11,12]. Previous work has shown that periosteal mesenchymal stem cells derived from oral tissues display an intrinsic tendency toward osteogenic differentiation. In a long-term culture study without osteogenic supplements, Ceccarelli et al. [11] reported progressive upregulation of ALP, BMP-2, RUNX-2, periostin, and osteoprotegerin, with expression levels at 45 days exceeding baseline and approaching those obtained under osteogenic induction. The parallel increase in ALP enzymatic activity further confirmed a time-dependent commitment toward the osteoblastic lineage. These findings support the view that periosteal MSCs naturally shift toward an osteoblast-like phenotype over prolonged culture [11]. Moreover, periosteal mesenchymal stem cells derived from craniofacial regions display higher turnover rates, elevated levels of bone markers, and a slower senescence process compared to periosteal mesenchymal stem cells derived from appendicular skeletal tissues such as the tibia [6].

The robust osteogenic potential and distinct physiological profile of periosteum-derived mesenchymal stem cells, when compared to other mesenchymal cell sources, may offer unique advantages in contexts where bone homeostasis is compromised, such as under microgravity conditions encountered during spaceflight. With the increasing emphasis on astronaut health during long-duration space missions, space biology research has increasingly focused on health issues associated with microgravity [13,14,15]. In terms of bone tissue, microgravity is associated with increased bone loss and bone resorption, which in turn elevates fracture risk [16]. This response is mainly due to a decrease in both the number and function of osteoblasts involved in bone formation, with reduced glucose utilization, reduced prostaglandin synthesis, and significantly altered actin cytoskeleton in osteoblasts [17]. However, studies in microgravity have traditionally focused exclusively on MSCs or osteoblasts derived from bone tissue (endosteum/bone marrow), without investigating the performance of MSCs derived from the periosteum.

Given that microgravity exposure has been associated with osteoporosis-like bone loss, primarily due to reduced mechanical loading on the skeleton [18], our group hypothesized that osteoblast-like cells from tissues less exposed to mechanical load may exhibit different responses from those located at high-load-bearing areas. Since the periosteum is a membrane, containing MSCs closely associated with bone tissue but not embedded within it, and lacks structural or mineralized components, it is conceivable that periosteal MSCs may be more resistant to the deleterious effects of reduced gravity.

Periosteum and endosteum, both considered primary sources of bone-forming cells during regeneration [19], occupy different compartments separated by the cortical bone, thus contributing differently to bone physiology, maintaining distinct cell pools [20]. Based on the principle of homeostasis between correlated tissues, where cells from one tissue often compensate for the deficit of an adjacent tissue, it is plausible to speculate that periosteal osteogenically induced cells may even exhibit enhanced osteogenic potential under reduced gravity conditions to counterbalance damage to endosteal cells. If the periosteum responds to external challenges (such as reduced gravity) in a similar manner by promoting mineralization, it could also serve as a foundation for developing new drugs to treat bone disorders. This hypothesis is supported from evidence that the periosteum can thicken, potentially leading to increased underlying cortical bone, in response to traumata [21,22], infection/inflammation [23], and pharmacological stimuli [24], indicating that it is an extremely reactive tissue. Although numerous studies have evaluated the behavior of endosteal and bone marrow-derived cells under microgravity conditions, to date, no publications have investigated human periosteal cells in this context.

Therefore, if the hypothesis that culturing P-MSCs under simulated microgravity enhances their osteogenic potential is confirmed, a new line of research may emerge for treating bone disorders such as osteoporosis, which affects one in ten individuals over the age of 50 in the United States [25]. Furthermore, considering the significant loss of bone density experienced by astronauts during prolonged spaceflight [16], using the environmental stressor of simulated or actual microgravity conditions as a potential tool for bone regeneration may represent a fascinating dichotomy. Despite the acknowledged importance of the periosteum in bone metabolism and repair, and the detrimental effects of reduced gravity on bone density [26], the scarcity of studies evaluating the behavior of human P-MSCs under microgravity drew our research group’s attention. This study aimed to evaluate human P-MSCs, cultured under simulated microgravity conditions.

## 2. Materials and Methods

### 2.1. Periosteum-Derived Mesenchymal Stem Cell Culture

Adult mesenchymal stem cells from a single donor were isolated from the palatal periosteum, cultured, validated, and cryopreserved by R-Crio Criogenia S.A. (Campinas, SP, Brazil), following a previously published methodology by our group [12]. In that study, periosteum-derived MSCs from 10 independent donors were fully characterized, including morphology, immunophenotyping, viability, and osteogenic differentiation capacity. The donor used in the present experiment was one of the 10 donors from that validated cohort and therefore had a previously established biological profile. As shown in the earlier publication, these cells exhibited a canonical MSC immunophenotype (CD105^+^, CD73^+^, CD90^+^, CD45^−^) and were able to differentiate into the osteoblastic lineage after 21 days in osteogenic medium, confirmed by Alizarin Red staining [12]. The use of this same cryopreserved cell stock ensures that the donor included in this study is consistent with the reproducible characteristics observed across the broader multi-donor cohort.

Cells were cultured in basal conditions for 49 days and seeded at a density of 110 cells/mm^2^ in 25 cm^2^ culture flasks (Sarstedt, Hildesheim, Germany) containing low-glucose DMEM (Life Technologies Corporation, Carlsbad, CA, USA) supplemented with 10% fetal bovine serum (Life Technologies Corporation, Carlsbad, CA, USA), 100 IU/mL penicillin, 50 μg/mL streptomycin (Sigma, St Louis, MO, USA), and 100 μM ascorbic acid (Sigma, St Louis, MO, USA). The culture medium was replaced every two days, and culture progression was monitored using an inverted phase-contrast microscope until cells reached 80% of confluence.

### 2.2. Osteogenic Differentiation

Cells were plated at a density of 110 cells/mm^2^ in DMEM supplemented with 10% FBS, 1% penicillin/streptomycin, 7 mM β-glycerophosphate, 0.1μM dexamethasone, and 50 μM ascorbic acid (Sigma, St Louis, MO, USA), and maintained for 14 days. The culture medium was replaced every two days.

### 2.3. Study Timeline

**Figure 1:**
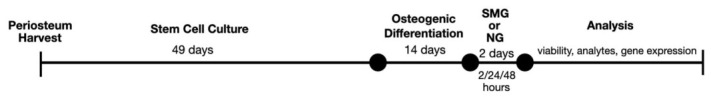
Schematic representation of the timeline used in this study.

Immediately after the osteogenic differentiation, the cells were maintained in unit gravity (normogravity—NG) or maintained in simulated microgravity conditions (SMG) for 3, 24 or 48 h. The cells were then examined for changes in cell viability, analytes and gene expression. The image below illustrates the timeline used in this study (Figure 1).

### 2.4. Simulated Microgravity (SMG) Conditions

**Figure 2:**
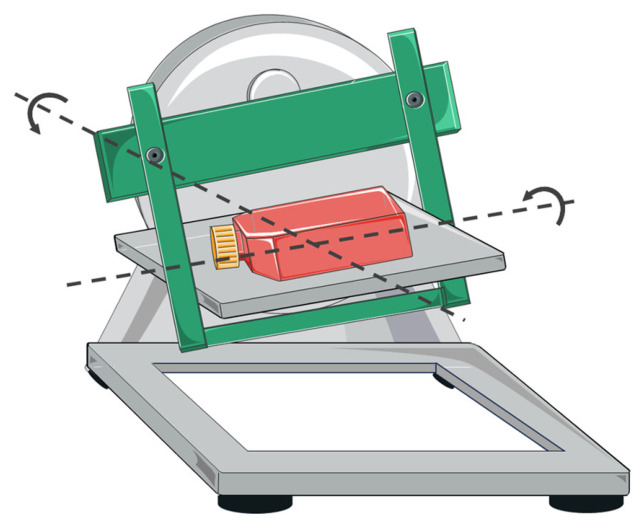
Schematic representation of the 3D clinostat (Random Positioning Machine). The system continuously rotates samples around multiple axes to randomize the gravity vector, thereby generating a simulated microgravity environment (<10^−3^ g). Cell culture flasks remain centered in the device to minimize shear forces, with rotation speed and orientation controlled to prevent sedimentation and directional gravitational cues. The figure was drawn using pictures provided by Servier Medical Art (https://smart.servier.com), licensed under CC BY 4.0 (https://creativecommons.org/licenses/by/4.0/), accessed on 2 October 2025.

Microgravity was simulated using a 3D clinostat RPM 2.0 (Yuri Gravity, Meckenbeuren, Germany) at the Space Life Sciences Lab (SLSL—Merritt Island, FL, USA). Culture flasks were carefully filled with medium, ensuring no air bubbles formed to avoid fluid shear stress. Flasks were sealed with a lid without a filter, to prevent leakage, which prevented gas exchange during the experiment. The flasks were secured on the device platform and then placed in a CO_2_ incubator (Thermo Fisher, Waltham, MA, USA). P-MSCs were exposed to a SMG environment (<10^−3^ g) for up to 48 h at 37 °C. As a control, P-MSCs were maintained under unit gravity (1 g) conditions (Normogravity, NG) (Figure 2).

The microgravity simulation was performed for just 48 h because the medium must be changed every 48 h. Studies of more than 48 h would require stopping the clinostat for a medium change resulting in the cells reacclimating to unit gravity (normogravity).

### 2.5. Cell Viability

P-MSCs cultured under different conditions were assessed for cell viability using the MTT assay. At 3, 24, and 48 h after cell plating, 10 μL of MTT solution (5 mg/mL) diluted in serum-free (DMEM Sigma, St Louis, MO, USA) was added to the treated cultures, which were then incubated for 3 h at 37 °C. After the incubation period, 100 μL of 10% DMSO (dimethyl sulfoxide, LGC, São Paulo, Brazil) solution was added. Following crystal solubilization, absorbance was measured at 590 nm using an ELX800 microplate reader (Epoch Biotek Instruments, Winooski, VT, USA). Optical density values were recorded. All experiments were performed in biological duplicates and technical triplicates for each time point.

### 2.6. Analysis of Osteogenesis-Related Analytes

At 3, 24 and 48 h, the supernatant was collected for both conditions (1 g and <10^−3^ g) and stored at −20 °C for subsequent analysis. All analyses were performed at the Interdisciplinary Center for Biotechnology Research (ICBR) at the University of Florida, FL, USA. Levels of 12 human bone-related biomarkers—adrenocorticotropic hormone (ACTH), dickkopf-1 (DKK-1), IL-6, insulin, leptin, tumor necrosis factor alpha (TNF-α), osteoprotegerin (OPG), osteocalcin (OC), osteopontin (OPN), sclerostin (SOST), IL-1β, and fibroblast growth factor 23 (FGF-23)—were measured using a fluorescent bead-based multiplex immunoassay with commercially available kits (Milliplex^®^ Human Cytokine/Chemokine/Growth Factor Panel A, HCYTA-60K, EMD Millipore Corporation, Billerica, MA, USA; Milliplex^®^ Human Bone Magnetic Bead Panel, HBNMAG-51K, EMD Millipore Corporation, Billerica, MA, USA) and a plate reader (Magpix^®^, EMD Millipore Corporation, Billerica, MA, USA), following the manufacturer’s instructions. Results were reported as total analyte concentration (pg/mL) per cell, highlighting differences between conditions and time points.

### 2.7. Gene Expression Analysis

Total RNA was extracted from cultured P-MSCs and prepared using the Illumina TruSeq^®^ Stranded mRNA Library Prep Kit (Illumina, San Diego, CA, USA), which included poly(A) selection for mRNA enrichment and incorporation of strand-specific information. The libraries were quantified, pooled, and sequenced on an Illumina NovaSeqX platform to generate paired-end 150 bp reads. Raw RNA sequencing data were processed using a standard RNA-seq pipeline as described below. Read quality was assessed using FastQC (v0.11.9), and low-quality reads and adapter sequences were trimmed using fastp. High-quality reads were then aligned to the GRCh38 human reference genome using the STAR aligner (v2.7.10a).

Gene quantification was performed using featureCounts to produce raw count matrices for each sample. Differential expression analysis was assessed using DESeq2, applying library size normalization and a negative binomial generalized linear model to identify statistically significant transcriptional changes between conditions.

Data visualization was conducted in R (version 4.5.1), including principal component analysis (PCA) to assess sample clustering, heatmap to depict global expression profiles, and volcano plot to highlight genes with significant expression changes.

### 2.8. Statistical Analysis

For the cell viability and analyte analysis, after conducting descriptive and exploratory data evaluations, and upon verification that assumptions for applying a general linear model were not met, generalized linear models were fitted to assess the main effects of group, time, and their interaction. All analyses were performed in R software, with a significance level of 5%.

For gene expression, differential expression analysis was performed using the DESeq2 package (v1.38.0) in R. Raw count matrices were normalized using DESeq2’s internal method, which estimates size factors to adjust for differences in library size and sequencing depth. A negative binomial generalized linear model was fitted for each gene to compare expression between experimental conditions at each time point. Resulting *p*-values were adjusted using the Benjamini–Hochberg method to correct for multiple comparisons and control the false discovery rate (FDR). Genes with an adjusted *p*-value (padj) < 0.05 and absolute log_2_ fold change (|log_2_FC|) > 2 were considered significantly differentially expressed.

## 3. Results

### 3.1. Cell Viability

**Figure 3:**
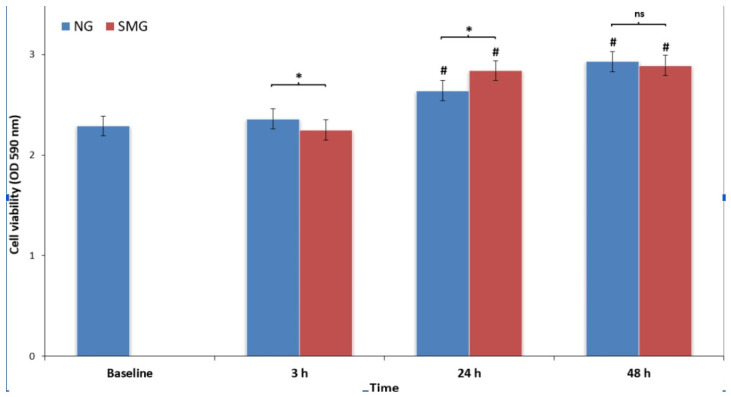
Cell viability of P-MSCs cultured under normogravity (NG, 1 g) and simulated microgravity (SMG, <10^−3^ g) evaluated by MTT assay at different time points. Bars represent standard deviation. # *p* ≤ 0.05 compared to baseline. * *p* ≤ 0.05 between experimental conditions at the same time point. ns = non significant (*p* > 0.05). Detailed quantitative values are provided in Supplementary Table S1.

Cell viability results for P-MSCs are presented in Figure 3. A decrease in cell viability was observed under simulated microgravity conditions (SMG, <10^−3^ g) compared to unit gravity (Normogravity, NG, 1 g) after 3 h of exposure. After 24 h, higher cell viability was detected under SMG compared to NG. At 48 h, no significant differences in cell viability were observed between the studied conditions.

### 3.2. Expression of Osteogenesis-Related Analytes

**Figure 4:**
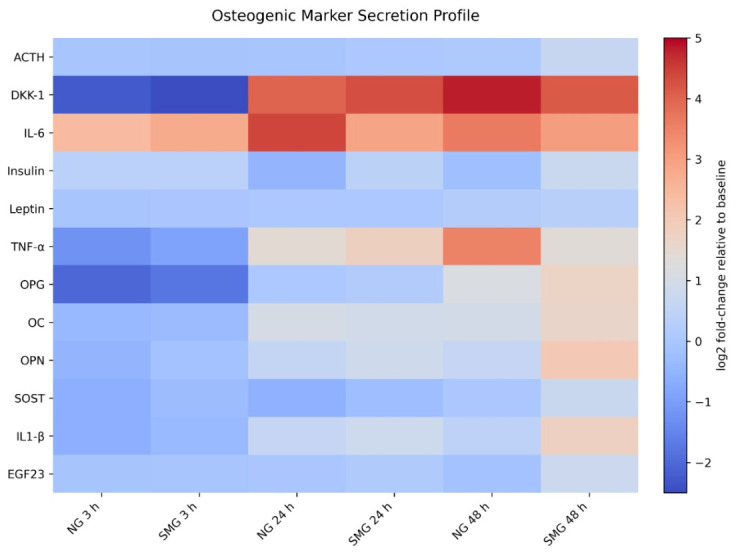
Heatmap representation of osteogenic marker secretion profiles in P-MSCs cultured under normogravity (NG, 1 g) and simulated microgravity (SMG, <10^−3^ g) conditions over time. Colors represent log2 fold-change relative to baseline concentrations for each analyte. Detailed quantitative values and statistical analyses are provided in Supplementary Table S2.

The osteogenic phenotype results are summarized in Figure 4, which presents a heatmap representation of osteogenic marker secretion profiles over time. Detailed quantitative values and statistical analyses are provided in Supplementary Table S1. P-MSCs exposed to SMG (<10^−3^ g) consistently exhibited higher levels of osteoprotegerin (OPG) at all time points compared to NG controls (1 g) (*p* < 0.05). At 24 and 48 h, insulin levels were also significantly increased under SMG (<10^−3^ g) compared to unit gravity (Normogravity, NG) (*p* < 0.05). By 48 h, cells in SMG (<10^−3^ g) showed a decreased secretion of DKK-1 and TNF-α, along with decreased interleukin-6 (IL-6) levels at both 24 and 48 h, relative to unit gravity (NG) (*p* < 0.05). There was no statistically significant difference between groups for ACTH, leptin, OC, OPN, SOST, IL-1β, PTH and FGF-23 (*p* > 0.05, data shown in Supplementary Table S1).

### 3.3. Gene Expression

**Figure 5:**
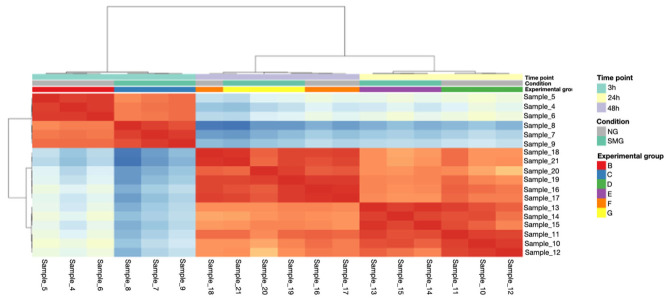
Heatmap of gene expression in P-MSCs across three time points under NG and SMG conditions. Samples were clustered based on the top 1000 most variable genes. The heatmap displays a correlation matrix among samples, with hierarchical clustering and associated dendrograms representing transcriptional similarity. Each column represents a sample, and rows represent genes. Sample groups are color-coded: B (red): NG at 3 h; C (blue): SMG at 3 h; D (green): NG at 24 h; E (purple): SMG at 24 h; F (orange): NG at 48 h and G (yellow): SMG at 48 h. Additional annotation bars indicate gravity condition (NG = gray; SMG = green) and time point (3 h = green; 24 h = yellow; 48 h = purple). NG = Normogravity; SMG = Simulated microgravity.

Heatmap analysis of the top 1000 most variable genes revealed dynamic transcriptional shifts across time points under SMG (Figure 5). From left to right, samples correspond to 3 h, 48 h, and 24 h. At 3 h, a distinct gene expression signature emerges, with a cluster of genes upregulated under SMG compared to NG, while another set was downregulated. By 24 h, the initially upregulated genes showed decreased expression, and the initially downregulated genes became upregulated, indicating a reversal in expression patterns. From 24 to 48 h, many of these same genes exhibited a second shift, suggesting a complex transcriptional adaptation process. These dynamic changes may reflect an early cellular response to microgravity that remodels or attenuates over time.

**Figure 6:**
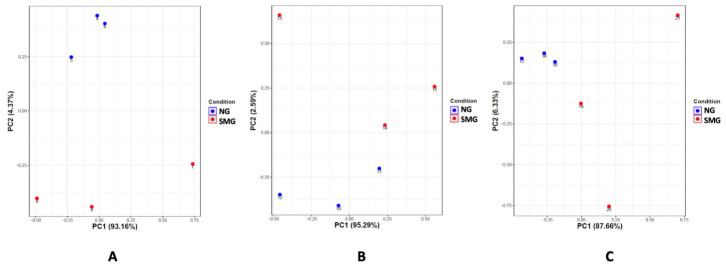
Principal component analysis (PCA) of transcriptomic profiles under simulated microgravity. Principal component analysis (PCA) was performed on normalized RNA-seq counts using the top 1000 most variable genes to compare normogravity (NG) and simulated microgravity (SMG) conditions at 3 h (**A**), 24 h (**B**), and 48 h (**C**). Each point represents an individual biological replicate. The percentage of variance explained by each principal component is indicated on the axes. Across all time points, PC1 explains the majority of the variance, indicating that experimental condition is the primary driver of global transcriptional differences.

Gene expression clustering patterns observed in the heatmap were further supported by Principal Component Analysis (PCA). At 3 h, PCA demonstrated a clear separation between NG and SMG samples, with PC1 accounting for 93.16% of the total variance. This trend continued at 24 h (PC1 = 95.29%) and remained evident at 48 h (PC1 = 87.66%), reinforcing the impact of SMG on the global transcriptional profile over time (Figure 6).

**Figure 7:**
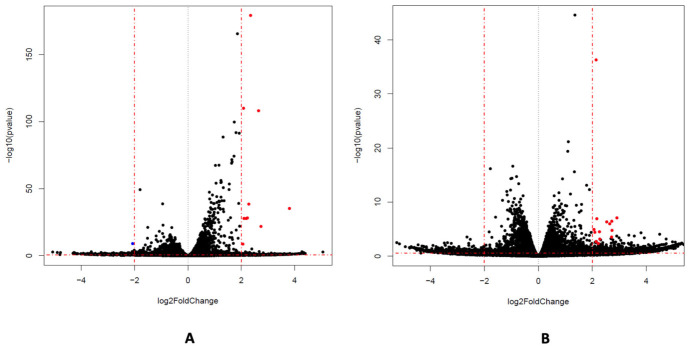
Differential gene expression under simulated microgravity conditions. Volcano plots showing differential gene expression between simulated microgravity and normogravity conditions at 3 h (**A**) and 24 h (**B**). The x-axis represents the log_2_ fold change (log_2_FC), and the y-axis represents the −log_10_(*p*-value). Vertical dashed red lines indicate the log_2_FC thresholds (|log_2_FC| ≥ 2), and the horizontal dashed red line indicates the significance threshold (*p* ≤ 0.05). Genes significantly upregulated under simulated microgravity are highlighted in red, whereas significantly downregulated genes are highlighted in blue. Because of the scale of the plot, the single downregulated gene in panel A appears close to the baseline and may be difficult to visualize. In (**A**) (3 h), the most prominently upregulated genes include IER3, HBEGF, IL6, BHLHE40, RGS16, IL11, LIF, JUNB, EGR2, and TNFSF9, while PDK4 was downregulated. In (**B**) (24 h), upregulated genes include MMP3, IL24, CH25H, RASD2, FAM133C, IL1RN, TDO2, TEC, WNT2, and CXCL6. No genes met the significance criteria for differential expression at 48 h (Group F–G), and therefore no volcano plot is shown for that time point.

Volcano plots highlighted several differentially expressed genes at 3 h, followed by fewer at 24 h, indicating a strong but transient transcriptional response to SMG (Figure 7). At 48 h, no volcano plot is shown because no genes met the differential expression criteria (padj < 0.05 and |log_2_FC| > 2). This likely reflects a stabilization of the transcriptional response after earlier fluctuations, suggesting that P-MSCs may reach a transient adaptive state.

In all timepoints GREM1, a BMP antagonist involved in skeletal development and osteoblast differentiation, was upregulated. MMP3, which contributes to extracellular matrix degradation and bone remodeling, also showed increased expression in all timepoints. In contrast, SCUBE3, a BMP pathway modulator, and PDGFC, a growth factor linked to mesenchymal cell recruitment and tissue repair, were downregulated in all timepoints. SMURF2, a regulator of TGF-β/BMP signaling, whose deficiency results in reduced bone mass, was also more highly expressed under SMG in all timepoints. Some key osteogenesis related genes (collagen, RUNX2, SOX9, BMP2, TGF-β and FGF) were found to be more highly expressed under SMG at least one of the three evaluated time points (collagen1, SOX9, BMP2, TGF- βs1/2/3 at 3 h; FGF 18 at 24 h and RUNX2 at 48 h).

At 3 h, SMG induced a pronounced transcriptional response in P-MSCs, characterized by the upregulation of genes associated with cellular stress, inflammation, and early differentiation. Transcription factors EGR1 (log_2_FC = 3.59), EGR2 (log_2_FC = 4.89), EGR3 (log_2_FC = 3.03), and JUNB (log_2_FC = 4.24) were significantly upregulated. Increased expression was also observed for inflammatory mediators IL6 (log_2_FC = 4.17), IL11 (log_2_FC = 4.48), LIF (log_2_FC = 3.95), and TNFSF9 (log_2_FC = 3.34). Genes involved in growth signaling and cellular proliferation, including HBEGF (log_2_FC = 3.64) and RGS16 (log_2_FC = 4.14), were also upregulated. MMP3 (log_2_FC = 2.17) and PLAU (log_2_FC = 3.62), associated with extracellular matrix remodeling, showed increased expression, as did the chromatin regulator KDM6B (log_2_FC = 2.92).

At 24 h, SMG modulated the expression of genes associated with inflammation, matrix remodeling, and growth signaling. Pro-inflammatory mediators IL24 (log_2_FC = 5.61) and IL1RN (log_2_FC = 4.89) were significantly upregulated, along with CXCL6 (log_2_FC = 5.37), a chemokine involved in tissue remodeling. MMP3, a matrix metalloproteinase, also showed sustained upregulation (log_2_FC = 4.31), consistent with continued extracellular matrix remodeling activity. FGF18, a fibroblast growth factor implicated in osteoblast differentiation, was upregulated with a log_2_FC of 2.69. WNT2, also involved in osteoblast differentiation, was upregulated with a log_2_FC of 2.26. Downregulated genes included GAS2L3 (log_2_FC = −3.19), associated with cytoskeletal regulation and mitosis.

At 48 h, SMG elicited a reduced transcriptional response in P-MSCs compared to earlier time points (3 h and 24 h). No differentially expressed genes associated with osteogenic signaling, matrix remodeling, or inflammatory pathways were detected at this time point, suggesting a potential stabilization or adaptation of the transcriptional program following prolonged microgravity exposure.

The combined transcriptional profile suggests that simulated microgravity triggers both an early stress-associated response and modulation of genes linked to osteogenic regulatory pathways, consistent with cellular adaptation during the short exposure window evaluated.

**Figure 8:**
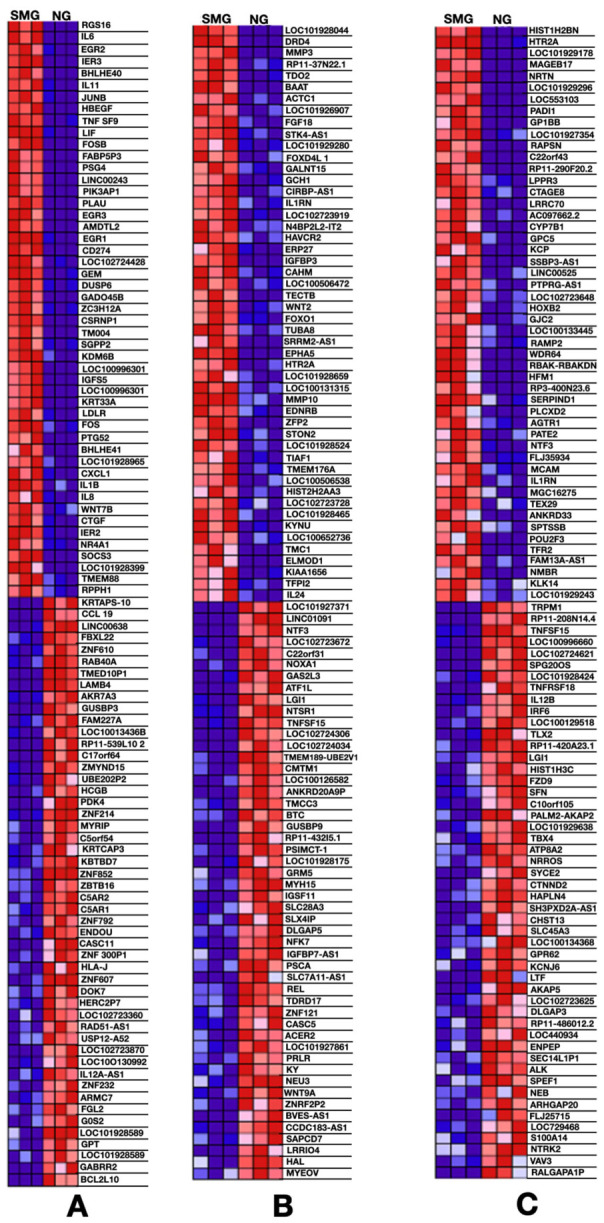
Hallmark gene set enrichment under simulated microgravity. Heatmaps showing the enrichment of Hallmark gene sets obtained by gene set enrichment analysis (GSEA) comparing simulated microgravity (SMG) and normogravity (NG) conditions at 3 h (**A**), 24 h (**B**), and 48 h (**C**). Each column represents an individual replicate, and rows correspond to significantly enriched gene sets. Gene sets were derived from ranked gene lists generated from the same dataset used for PCA. Color intensity reflects normalized enrichment scores (NES), with red indicating positive enrichment and blue indicating negative enrichment under SMG relative to NG.

Gene Set Enrichment Analysis (GSEA) of hallmark gene sets revealed time-dependent transcriptional modulation in response to SMG. At 3 h, SMG led to robust enrichment of pathways associated with inflammation (e.g., TNF-α signaling via NF-κB, IL6/JAK/STAT3 signaling), apoptosis, and hypoxia, indicating an early activation of stress-responsive and immune signaling programs (Figure 8A). By 24 h, similar pathways remained enriched, although the overall magnitude of enrichment was attenuated, suggesting partial resolution or regulation of the initial response (Figure 8B). At 48 h, gene set enrichment was markedly diminished, and clustering between NG and SMG groups was less distinct, reflecting reduced transcriptomic divergence and supporting the interpretation of transcriptional adaptation or stabilization over prolonged exposure (Figure 8C). These pathway-level findings reinforce the temporal dynamics observed in the gene-level differential expression analyses.

## 4. Discussion

Literature on the effects of simulated microgravity (SMG) on periosteum-derived MSCs and/or osteoblasts is scarce. However, there is some data showing that periosteum stem cells are more resistant to acute stress than bone marrow stem cells and, in some circumstances, may promote bone marrow regeneration and the support of hematopoiesis [27]. Our preliminary data suggests that the response of the periosteal cells to the microgravity stress may make them more useful for use in bone tissue engineering in a microgravity environment than stem cells from other sources.

This is the first study to investigate the behavior of human P-MSCs cells under simulated microgravity. Nonetheless, our findings are consistent with and extend prior observations in animal models. Liu et al. (2023) [28] showed that rat periosteal stem cells exhibit an enhanced osteogenic profile under simulated microgravity, with significant upregulation of RUNX2, SP7 (Osx), COL1A1, OCN, OPN and BMP2 both in vivo and in vitro. Their results also suggested that periosteal stem cell activation may help maintain cortical bone stability during mechanical unloading. In line with these findings, our data indicate that human periosteal cells also respond to simulated microgravity with measurable changes in viability and osteogenic behavior, although the directionality and magnitude of these effects may differ between species and experimental systems. The emerging picture across human and rodent studies is that periosteal cells play a critical role in cortical bone homeostasis under altered mechanical environments. This interpretation is further supported by large-scale anthropological evidence: a recent analysis of more than 9000 years of European skeletal material reported that human physiology tends to compensate for age-related trabecular bone loss through subperiosteal bone deposition, albeit insufficiently [29]. Together, these data reinforce that periosteal progenitors are highly responsive to mechanical cues and may help mitigate the structural consequences of bone loss. Our study extends the understanding of periosteal biology in the context of microgravity and provides translational insight that complements previous animal work by showing how human periosteum-derived MSCs react to microgravity simulation.

It is well established that bone marrow-derived mesenchymal stem cells and periosteum-derived mesenchymal stem cells contribute differently to bone maintenance and repair. Different types of bone injuries are repaired by osteoprogenitor cells from distinct sources: periosteal cells are primarily responsible for regenerating the bone stroma and marrow following unstable fractures, whereas bone perforation injuries are mainly repaired by bone marrow-derived osteoprogenitor cells [30]. Since microgravity can be viewed as a form of tissue/cellular destabilization caused by the reduction in gravitational force, we hypothesized that P-MSCs might play a more prominent role in osteogenesis under microgravity when compared to endosteal or bone marrow cells. Although the literature includes studies reporting a reduction in osteogenic potential in pre-osteoblasts derived from bone tissue [31,32] and from bone marrow [33,34] under microgravity, there was a complete lack of studies assessing P-MSCs in this context.

This study evaluated the early cellular and molecular responses of periosteum-derived MSCs exposed to simulated microgravity (exposure times from 3 to 48 h). Therefore, functional osteogenic assays such as ALP activity or mineralization, which require longer induction periods, were not included. Thus, the presented data primarily reflect early regulatory signaling (molecular adaptations) rather than definitive functional differentiation.

Although the viability of P-MSCs cells was reduced after 3 h under SMG, these cells showed higher viability than those in normogravity after 24 h. At 48 h, no significant difference in viability was observed between the two conditions. Moreover, evident cell growth occurred within the 48 h period for both normogravity and microgravity groups (see Figure 3). This behavior contrasts with that of osteoblasts derived from bone and bone marrow cultured under microgravity, which typically show a marked reduction in viability [17,33].

Analysis of osteogenesis-related analytes showed that culturing P-MSCs under microgravity increased insulin secretion, which is known to support bone formation and maintenance. Beyond its role in glycemic control, insulin directly affects osteoblasts by promoting anabolic activity and facilitating bone matrix deposition. It plays a critical role in bone homeostasis by modulating the balance between bone formation and resorption [35]. Individuals with insulin resistance or diabetes often exhibit impaired bone density and metabolism, significantly increasing their risk of fractures and other skeletal complications [36].

Microgravity also increased the secretion of osteoprotegerin (OPG), which plays a key role in regulating bone resorption by acting as a decoy receptor for RANKL (Receptor Activator of Nuclear Factor-κB Ligand), thereby blocking osteoclast activation [37]. By maintaining the balance between bone formation and resorption, OPG is critical for preventing conditions such as osteoporosis, in which resorption exceeds formation [38]. The present results contrast sharply with findings from bone-derived osteoblasts, where microgravity has been shown to reduce OPG secretion [32], and to impair the regulation of bone resorption by osteoclasts [18]. Supporting the anabolic effect of P-MSCs in microgravity, the observed reduction in DKK1 and TNF expression contributes to enhanced bone regeneration, since both molecules are known inhibitors of the Wnt signaling pathway, which regulates osteoblast differentiation and function [39].

Furthermore, IL-6 secretion was reduced after 24 and 48 h of exposure to microgravity. IL-6 is a pro-inflammatory cytokine with pleiotropic effects on immune responses, including the activation of immune cells and the induction of growth factor secretion [40]. It also plays a key role in bone homeostasis by stimulating RANKL production in bone marrow stromal cells, thereby promoting bone resorption [41]. Therefore, the reduction in IL-6 observed in this study may represent a protective mechanism against bone resorption by the periosteal cells. Again, these findings contradict previous studies with bone-derived osteoblasts from other sources, in which microgravity increased IL-6 secretion [32,42].

PCA, volcano plots, and heatmaps demonstrated that SMG exerted an important influence on gene expression compared to normogravity. The results at 3 h revealed an immediate cellular response to microgravity, including changes in stress-related, inflammatory, and metabolic genes. Early activation of stress-response pathways suggests that cells rapidly detect and react to the new environment. The 24 h results show sustained effects and by 48 h, cells appeared to have reached a stable state under microgravity. The absence of reduced cell viability at this time point rules out extensive cell death. Thus, PCA, volcano plot, and heatmap data suggest that microgravity induces a rapid gene expression response—initiated as early as 3 h—which persists at 24 h but diminishes significantly by 48 h, implying cellular adaptation or differentiation into a new microgravity-adapted phenotype. Although transcriptional stabilization at 48 h is consistent with the transient nature of early responses to simulated microgravity, alternative explanations—including donor-specific patterns or limited sensitivity to detect subtle changes at later time points—cannot be excluded.

These gene-level observations were further supported by pathway-level analysis using Gene Set Enrichment Analysis (GSEA). At 3 h, hallmark gene sets associated with inflammatory signaling (e.g., TNF-α via NF-κB, IL6/JAK/STAT3), apoptosis, and hypoxia were highly enriched in SMG-exposed cells, reflecting early stress and immune activation. By 24 h, many of these pathways remained active but showed attenuated enrichment, suggesting partial adaptation. At 48 h, enrichment patterns diminished, indicating transcriptional stabilization under prolonged microgravity. This temporal shift in pathway activation aligns with previous findings showing that early stress and inflammatory responses often precede functional adaptation in stem or progenitor cells under altered mechanical environments [43].

RNA-seq analysis revealed time-dependent regulation of key osteogenic regulators in P-MSCs exposed to SMG. At 3 h, genes such as Sox9, BMP2, TGF-βs1/2/3, and multiple collagen isoforms were significantly upregulated, suggesting early commitment toward osteogenic differentiation. At 24 h, FGF18, a known stimulator of osteoblast proliferation, showed increased expression, and by 48 h, RUNX2 was upregulated, indicating progression toward later stages of osteogenic programming. These results differ from previous studies reporting suppression of osteogenic genes under microgravity [44,45] and instead support a model in which periosteal cells retain and even enhance osteogenic potential under these conditions.

Under simulated microgravity, the elevated expression of Sox9, BMP2, TGF-β1, TGF-β2, and collagen-related genes at 3 h suggests that P-MSCs begin early maturation programming, whereas osteogenesis-related protein secretion was detected only at 24 and 48 h (see Figure 4, Table S2). The continued upregulation of collagen genes at 24 and 48 h, and of Runx2 and FGFs at 48 h, may indicate sustained osteogenic stimulation, correlating with increased insulin secretion, decreased IL-6, and significant downregulation of DKK-1 and TNF—all linked to osteogenesis-promoting effects. The upregulation of osteogenesis-related pathways and markers GREM1, SMURF2 and MMP3 were also verified after simulated microgravity stimulus.

RNA-seq data also clearly show an immediate stress response at 3 h, with upregulation of inflammatory and metabolic genes (IER3, HBEGF, IL6, BHLHE40, RGS16, IL11, LIF, JUNB, EGR2, TNFSF9, PDK4). By 24 h, cells appear to engage in regulatory adjustments and adaptation. Although IL-6 gene expression was elevated at 3 h, suggesting activation of the NF-κB inflammatory pathway, protein-level analysis (Figure 4, Table S2) revealed no difference in IL-6 between groups at this time point and, in fact, a significant decrease at 24 and 48 h. We hypothesize that this discrepancy may be due to rapid cytokine translation and degradation or limited early-phase regulation. However, additional experimental validation is required. At 24 h, increased MMP-3 expression suggests extracellular matrix remodeling and possible modulation of osteogenic differentiation. No osteogenesis-related genes or inflammatory markers were significantly regulated at this time point. This could be related to cellular transcriptional stabilization or adaptation following the initial response. It could also result from limited statistical power or overly stringent thresholds for differential expression.

In summary, the results of this study indicate that P-MSCs exhibit an initial adaptive response to SMG involving inflammatory signaling and extracellular matrix remodeling, potentially supporting osteogenesis. The data support the hypothesis that these cells develop osteogenesis-favoring molecular programs under microgravity, transitioning from an inflammatory to a pro-osteogenic profile. However, it is important to state that we used periosteum cells derived from the palate and that periosteum cells from other sites might have different characteristics. Under microgravity conditions, bone mineral density decreases in the lower extremities but increases in the skull [46]. Moreover, it is also known that periosteal mesenchymal stem cells from craniofacial regions display higher turnover rates, elevated levels of bone markers, and a slower senescence process compared to P-MSCs derived from appendicular skeletal tissues such as the tibia [6]. Further studies are needed to validate these findings, to strengthen the proposed hypothesis, and to clarify how periosteum-derived osteoprogenitor cells and bone regeneration processes behave in vivo under prolonged microgravity conditions. Furthermore, since hormonal signals and complex tissue interactions may affect cellular responses in living systems, in vivo studies are required to compliment cellular studies.

A limitation of this study is the use of cells from a single donor. However, this donor was previously included in a 10-donor periosteum MSC characterization study by our group [12], in which all donors exhibited comparable morphology, MSC immunophenotype, viability, and osteogenic differentiation capacity. The donor used here matched this established profile, indicating that the observed responses are consistent with a broader and validated biological baseline. Although the donor used in this study had been previously characterized as part of a larger donor cohort published by our group, this does not fully overcome the limitations associated with the use of a single donor, particularly in the context of transcriptomic analyses, which may be strongly influenced by donor-specific biological variability. In addition, the study presents limited statistical power due to the low number of biological replicates. Even so, we emphasize that the present work represents a mechanistic proof-of-concept, and future experiments involving multiple donors are needed to assess inter-individual variability under simulated microgravity.

It is also important to state that clinostat-based models have inherent limitations, including residual acceleration, shear forces, and continuous medium mixing, which can influence nutrient and gas exchange and contribute to mechanical artifacts not presented in true microgravity. Although RPM platforms are widely used and valuable for studying mechanobiological responses, they simulate microgravity by randomizing the gravity vector rather than eliminating gravitational forces and cannot fully reproduce the biomechanical environment experienced during orbital spaceflight [47]. Moreover, the lack of gas exchange in sealed culture flasks and the potential influence of altered nutrient diffusion may affect cellular responses. All these factors could contribute to the pronounced early inflammatory and stress-related transcriptional signatures observed in the study.

Another limitation of this study was the simulation of microgravity for no longer than 48 h. This is a limitation inherent in the experimental model, as the medium must be changed every 48 h. Study of more than 48 h would require stopping the clinostat for medium change, exposing the cells to normogravity. Maintenance of stem cells for hours (or couple days), and not weeks, is common in the literature studying stem cells in simulated microgravity [48,49]. Evaluation of periosteal cells under real microgravity (e.g., at the ISS) for extended periods of time is vital for establishing these cell’s potential utility for bone regeneration in microgravity.

If confirmed in vivo, our findings might open new avenues for the treatment of bone diseases such as osteoporosis using the secretome of periosteal cells. Additionally, during long-term space missions, periosteal cell cultures derived from astronauts themselves might potentially be used to mitigate microgravity-induced bone loss. These translational and clinical implications, however, need to be presented as first, preliminary and hypothesis-generating issues.

## 5. Conclusions

The aim of this study to evaluate the behavior of periosteum derived mesenchymal stem cells cultured under a simulated microgravity environment was achieved. Enhanced pro-osteogenic regulatory modulation was demonstrated.

Increased osteoprotegerin (OPG) levels together with decreased DKK-1 and TNF, and reduced IL-6 at later time points, collectively support a shift toward pro-osteogenic signaling. In combination with the observed regulation of osteogenesis-related genes (including pathways such as BMP, WNT, and RUNX2), these findings suggest a transition from an early stress response toward a phenotype potentially favorable for osteogenic differentiation occurs in simulated microgravity.

The limitations of this study include the limited number of donors and short time in simulated microgravity conditions. These may be overcome by future studies using a higher number of biologic samples and performing studies in real microgravity (e.g., at the ISS).

## 6. Patents

The patent number BR 10 2025 011426 7 was a result of this research and might be used for the establishment of new drugs to treat bone diseases.

## Data Availability

The original contributions presented in this study are included in the article. Further inquiries can be directed to the corresponding author.

## Supplementary Materials

The following supporting information can be downloaded at: https://www.mdpi.com/article/10.3390/cells15110989/s1. Table S1. Mean (standard deviation) of cell viability measured by MTT assay (absorbance values) for osteoblast-like cells, according to experimental condition and time point. Table S2. Mean and standard deviation of analyte concentrations (pg/mL) associated with the osteogenic phenotype measured by Luminex assay in osteoblast-like cells cultured under normogravity (1 g) and simulated microgravity (<1 g) conditions.
