# Supplementary material for: Enhanced Pro-Osteogenic Regulatory Modulation in Mesenchymal Stem Cells Derived from the Periosteum Under Simulated Microgravity
Source: Cells. 2026 May 28;15(11):989. doi: 10.3390/cells15110989 (PMC13256461; doi:10.3390/cells15110989)
Supplement: Supplementary file 1 [file cells-15-00989-s001.zip › cells-4315702-supplementary.pdf]

Supplementary Table S1. Mean (standard deviation) of cell viability measured by MTT assay (absorbance values) for osteoblast-like cells, according to experimental condition and time point.

Time	Experimental Condition	
	Normogravity (1g)	Microgravity (<1g)
	Mean (SD)	Mean (SD)
Baseline	2.29 (0.19)	-
3 hours	2.36 (0.06) Ac	2.25 (006) Bb
24 hours	*2.64 (0.06) Bb	*2.84 (0.11) Aa
48 hours	*2.93 (0.07) Aa	*2.89 (0.14) Aa

*Different from the group evaluated in baseline (p≤0.05). p(group)=0.8495; p(time)<0.0001; p(interaction)=0.0002. Different letters (uppercase horizontally and lowercase vertically) indicate statistically significant differences (p≤0.05).

Supplementary Table S2. Mean and standard deviation of analyte concentrations (pg/mL) associated with the osteogenic phenotype measured by Luminex assay in osteoblast-like cells cultured under normogravity (1g) and simulated microgravity (<1g) conditions.

Variable	Time	Experimental Condition	
		Normal gravity	Simulated microgravity
		Mean (SD)	Mean (SD)
ACTH	Baseline	2.26 (0.03)	-
	3 hours	2.22 (0.06) Aa	2.18 (0.02) Aa
	24 hours	2.20 (0.17) Aa	2.36 (0.09) Aa
	48 hours	2.41 (0.10) Aa	*3.56 (2.24) Aa
p-values		p(group)=0.2853; p(time)=0.1228; p(interaaction)=0.4107; p(control group)=0.0469	
DKK1	Baseline	26.68 (3.71)	-
	3 hours	*5.62 (0.40) Ac	*4.89 (0.80) Ab
	24 hours	*420.40 (13.64) Ab	*514.41 (113.42) Aa
	48 hours	*741.15 (18.41) Aa	*468.87 (59.53) Ba
p-values		p(group)=0.0256; p(tempo)<0.0001; p(interação)=0.0002; p(grupo controle)<0.0001	
IL6	Baseline	26.25 (2.21)	-
	3 hours	*138.08 (24.43) Ac	*178.24 (17.58) Aa
	24 hours	*558.54 (229.98) Aa	*195.04 (20.69) Ba
	48 hours	*325.90 (37.40) Ab	*214.40 (26.66) Ba
p-values		p(group)=0.0243; p(time)<0.0001; p(interaction)=0.0003; p(control group)<0.0001	

Insulin	Baseline	80.37 (22.85)	-
	3 hours	104.30 (1.97) Aa	104.73 (1.29) Aa
	24 hours	*58.56 (13.54) Ba	107.03 (2.94) Aa
	48 hours	69.92 (21.25) Bb	*135.86 (48.08) Aa
p-values		p(group)<0.0001; p(time)=0.0378; p(interaction)=0.0055; p(control group)=0.0005	
Leptin	Baseline	35.37 (0.51)	-
	3 hours	34.82 (0.63) Ac	35.33 (0.41) Ac
	24 hours	36.64 (1.17) Ab	36.64 (0.55) Ab
	48 hours	*41.26 (1.36) Aa	*43.32 (1.77) Aa
p-values		p(group)=0.1255; p(time)<0.0001; p(interaction)=0.3383; p(control group)<0.0001	
TNF	Baseline	0.28 (0.13)	-
	3 hours	0.12 (0.00) Ab	0.15 (0.00) Aa
	24 hours	0.76 (0.08) Ab	*0.99 (0.09) Aa
	48 hours	*3.19 (1.14) Aa	0.72 (0.04) Ba
p-values		p(group)=0.0148; p(time)<0.0001; p(interaction)<0.0001; p(control group)<0.0001	
OPG	Baseline	1,885.17 (32.08)	-
	3 hours	*467.55 (30.63) Bc	*562.36 (43.33) Ac
	24 hours	1,970.55 (159.59) Bb	2,144.50 (429.76) Ab
	48 hours	*4,183.37 (322.27) Ba	*6,089.38 (1,333.15) Aa
p-values		p(group)=0.0418; p(time)<0.0001; p(interaction)=0.3798; p(control group)<0.0001	
Osteocalcin	Baseline	167.51 (35.33)	-
	3 hours	129.72 (22.24) Ab	134.05 (8.17) Ab
	24 hours	*343.23 (142.03) Aa	*324.61 (130.79) Aa
	48 hours	*328.20 (66.03) Aa	*525.74 (110.99) Aa
p-values		p(group)=0.5392; p(time)<0.0001; p(interaction)=0.3480; p(control group)<0.0001	
Osteopontin	Baseline	84.95 (28.28)	-
	3 hours	61.35 (43.56) Ab	78.59 (26.71) Ab
	24 hours	127.01 (38.21) Aa	152.74 (0.58) Aa
	48 hours	130.07 (59.78) Aa	*342.91 (106.00) Aa
p-values		p(group)=0.1263; p(time)=0.0013; p(interaction)=0.6046; p(control group)=0.0002	
SOST	Baseline	68.55 (16.23)	-
	3 hours	44.85 (5.91) Ab	56.07 (11.50) Ab
	24 hours	47.10 (6.40) Ab	58.37 (7.88) Ab
	48 hours	69.74 (36.39) Aa	*113.15 (60.78) Aa
p-values		p(group)=0.1027; p(time)=0.0109; p(interaction)=0.9724; p(control group)=0.0107	
IL1-beta	Baseline	0.23 (0.01)	-
	3 hours	0.15 (0.02) Ab	0.18 (0.01) Ab
	24 hours	0.35 (0.08) Aa	*0.41 (0.08) Aa
	48 hours	0.31 (0.02) Aa	*0.80 (0.82) Aa
p-values		p(group)=0.1555; p(time)=0.0004; p(interaction)=0.4864; p(control group)<0.0001	

EGF23	Baseline	5.72 (0.22)	-
	3 hours	5.49 (0.15) Aa	5.67 (0.38) Aa
	24 hours	5.69 (1.09) Aa	6.39 (0.28) Aa
	48 hours	5.26 (2.22) Aa	*9.89 (4.91) Aa
p-values		p(group)=0.0918; p(time)=0.4711; p(interaction)=0.2335; p(control group)=0.0286	

*Different from the group evaluated in baseline (p≤0.05). Different letters (uppercase horizontally and lowercase vertically) indicate statistically significant differences (p≤0.05).
